# Assessment of Antibacterial and Anti-biofilm Effects of Vitamin C Against *Pseudomonas aeruginosa* Clinical Isolates

**DOI:** 10.3389/fmicb.2022.847449

**Published:** 2022-05-20

**Authors:** Wedad M. Abdelraheem, Marwa M. M. Refaie, Rehab Kamal Mohamed Yousef, Aliaa S. Abd El Fatah, Yosra M. Mousa, Rabab Rashwan

**Affiliations:** ^1^Department of Medical Microbiology and Immunology, Faculty of Medicine, Minia University, Minya, Egypt; ^2^Department of Pharmacology, Faculty of Medicine, Minia University, Minya, Egypt; ^3^Department of Pathology, Faculty of Medicine, Minia University, Minya, Egypt; ^4^Internal Medicine Department, Faculty of Medicine, Minia University, Minya, Egypt; ^5^Chest Department, Faculty of Medicine, Minia University, Minya, Egypt

**Keywords:** antimicrobial and anti-biofilm effects of vitamin C, anti-biofilm strategies, synergism, MDR, biofilm former *P. aeruginosa* strains

## Abstract

There is a persistent need to look for alternative therapeutic modalities to help control the pandemic of antimicrobial resistance. Assessment of antibacterial and anti-biofilm effects of vitamin C (ascorbic acid) was the aim of the current study. The micro-dilution method determined the minimal inhibitory concentration (MIC) of ascorbic acid or antibiotics alone and in combinations against *Pseudomonas aeruginosa* (*P. aeruginosa*) clinical isolates. The micro-titer plate method monitored the effect of ascorbic acid on the biofilm-producing isolates of *P. aeruginosa*. The effect of ascorbic acid on the differential expression of different antibiotic-resistant genes and biofilm encoding genes of *P. aeruginosa* isolates were also tested using real-time polymerase chain reaction (PCR). For *in vivo* assessment of the antibacterial effects of ascorbic acid alone or combined with an antibiotic, rats were infected with *P. aeruginosa* clinical isolate followed by different treatment regimens. MICs of ascorbic acid among *P. aeruginosa* isolates were in the range of 156.2–1,250 μg/ml, while MIC50 and MIC90 were 312.5 and 625 μg/ml, respectively. At sub-inhibitory concentrations (19.5–312.5 μg/ml), ascorbic acid had 100% biofilm inhibitory effect. Furthermore, ascorbic acid-treated bacteria showed downregulation of genes underpinning biofilm formation and antibiotic resistance. *In vivo* assessment of vitamin C and ceftazidime in rats showed that administration of both at a lower dose for treatment of pseudomonas infection in rats had a synergistic and more powerful effect. Vitamin C shows excellent *in vitro* results as an antibacterial and anti-biofilm agent. Vitamin C should be routinely prescribed with antibiotics to treat bacterial infections in the clinical setting.

## Introduction

*Pseudomonas aeruginosa* (*P. aeruginosa*) is a versatile bacteria characterized by multiple resistance to various antimicrobial agents. It is one of the top causes of opportunistic infections and a primary causative agent of hospital-acquired infections (Stover et al., 2000). The emergence of multi-drug resistant (MDR) *P. aeruginosa* is a potential public health risk and may compromise effective antibiotic therapy. Biofilm formation is an essential cause of antibiotic resistance among *P. aeruginosa* clinical isolates. There are a list of factors considered to be responsible for biofilm resistance, which include restricted penetration of antimicrobials into a biofilm barrier, decreased growth rate of biofilm persister cells, and expression of possible biofilm-specific resistance genes (Lewis, 2001). Biofilm formation is leading to another form of resistance, also called tolerance or phenotypical resistance of biofilm embedded cells. Biofilm-forming *P. aeruginosa* has the ability to convert to an antibiotic-resistant phenotype after antibiotic exposure with enhanced antibiotic tolerance (Drenkard and Ausubel, 2002).

There is considerable concern about the pandemic increase in antibiotic-resistant bacteria leading to treatment failure (Hancock and Speert, 2000). Vitamin C (ascorbic acid) represents one such alternative, and many studies have demonstrated its antibacterial action. It has a strong growth inhibitory effect on *Staphylococcus aureus*, *Enterococcus faecalis* (Isela et al., 2013), *Helicobacter pylori*, *Campylobacter jejuni* (Zhang et al., 1997), and *Mycobacterium tuberculosis* (Vilchèze et al., 2013), and even on fungi such as *Aspergillus* (Gupta and Guha, 1941). At first, it was hypothesized that the antimicrobial effect of vitamin C was due to its pH-lowering effect. However, another study proved the potent antimicrobial properties of vitamin C directed against *Streptocccus pyogenes*, even in pH-neutral conditions. Also, vitamin C is a powerful antioxidant agent produced against free radicals and reactive oxygen species (Slade and Knox, 1950).

Vitamin C is cheap, available, and has few or no side effects. Vitamin C is frequently prescribed as a nutritional supplement, has known antioxidant effects, and has been used as an adjuvant in cancer chemotherapy. Vitamin C is a gluconic acid lactone derived from glucuronic acid and water-soluble ketolactone with two ionizable hydroxyl groups (Sorice et al., 2014). In nature, there are two isomeric molecules of vitamin C present in equal parts, the reduced form (D-ascorbic acid) and the chemically active and oxidized form (L-ascorbic acid) (Hong et al., 2016), which are mutually interchangeable. The natural supplies of vitamin C are citrus fruits, kiwi, mango, strawberries, papaya, tomatoes, green leafy vegetables, and broccoli (Farris, 2014). This study investigated the effect of ascorbic acid alone and in combination with antibiotics on the growth and biofilm-forming potential of clinical isolates of *P. aeruginosa in vitro.* We also examined the effect of ascorbic acid alone and in combination with an antibiotic on treating *P. aeruginosa* infection in rats.

## Materials and Methods

### Microorganisms

Previously identified *P. aeruginosa* clinical isolates collected from patients with burn or infected wounds, at the surgery department of Minia University Hospital, were included in this study. The identification of the isolates was done according to morphological characteristics and biochemical reactions using API Test Kits (bioMerieux, France). We selected 50 biofilm-producing *P. aeruginosa* from all other isolates to perform this study.

### Determination of Minimal Inhibitory Concentration of Ascorbic Acid or Antimicrobials Alone and in Combinations

The minimal inhibitory concentration (MIC) of ascorbic acid or antibiotics alone and in combinations against all isolates was determined by micro-dilution method, according to the clinical and laboratory standard institute (CLSI) guidelines [Clinical and Laboratory Standard Institute [CLSI], 2019]. A stock solution of ascorbic acid was prepared by dissolving commercially purchased ascorbic acid (L-ascorbic acid, Sigma) in sterile distilled water (20 mg/ml). Stock solutions of the following antibiotics: piperacillin, piperacillin/tazobactam, ceftazidime, ciprofloxacin, and gentamicin were prepared by dissolving commercially purchased antibiotics in sterile distilled water (1 mg/ml). Müeller-Hinton broth (MHB) (Difco, United States) was used as a basal medium. Serial dilutions of ascorbic acid or antibiotics alone and in combinations were prepared in 100 μl volume of MHB in micro-titer plate wells. A quantity of 10 μl of bacterial suspension of each isolate with 0.5 McFarland turbidity was then inoculated in the wells. Wells containing a basal medium with and without bacterial suspension, but free of antibiotic and ascorbic acid, and wells containing the antibiotic or ascorbic acid were included in each assay as growth control. The plates were incubated for 24 h at 37°C. The lowest concentration of antibiotics separately or combined with ascorbic acid, which prevented the growth, was regarded as the MIC. The MIC results were interpreted according to CLSI guidelines [Clinical and Laboratory Standard Institute [CLSI], 2019].

### Determination of Fractional Inhibitory Concentration

Fractional inhibitory concentration (FIC) was used to interpret the MIC results as follows (Mackay et al., 2000): FIC of antibiotic = MIC of the antibiotic combined with ascorbic acid/MIC of antibiotic alone. FIC ≤ 0.5 means syngergism, FIC > 0.5–4 means indifference, and FIC > 4 means antagonism.

### Time-Kill Kinetics Assay

Vitamin C was tested to detect the time-kill kinetics. Briefly, grown culture of MHB with pure bacterial colonies (1.0 × 10^6^ CFU/ml) was supplemented with ascorbic acid (625 μg/ml). Serial dilution was performed at each time point (0, 2, 4, 6, 12, and 24 h), and subculture on Muller Hinton agar plates was incubated at 37°C. Positive control test was performed for the tested strains without ascorbic acid. A graph of log CFU versus time was created. Each experiment was performed in triplicates (Appiah et al., 2017).

### Effect of Ascorbic Acid on Biofilm Production

The effect of ascorbic acid on the biofilm-producing isolates of *P. aeruginosa* was assessed by the microtiter plate method according to the instructions by Samet et al., 2013. Initially, 190 μl of bacterial suspension equivalent to 0.5 McFarland in Luria Bertani broth (LB) was inoculated in 96 microtiter plates. Sub-MIC of ascorbic acid was added to each well, excluding the positive and negative control wells. Plates were incubated at 37°C for 24 h. After incubation, the content of the wells were gently removed. The wells were washed with phosphate-buffered saline solution to remove free-unattached bacteria. Biofilms formed by adherent bacteria were air- and heat-fixed at 60°C for 1 h, and then stained with crystal violet (0.1%). Excess stain was rinsed off and the wells were washed with water. Ethanol 95% was added to the wells, and after 15 min, the optical densities (ODs) of the stained bacteria were determined with an ELISA reader (model CS, Biotec) at 590 nm. These OD values were considered an index of bacteria-forming biofilms. Experiments were performed in triplicate, and the data were then averaged.

### Effect of Ascorbic Acid on Relative Genes Expression

*Pseudomonas aeruginosa* isolates were tested to express different antibiotic-resistant genes and biofilm encoding genes before and after treating the isolates with ascorbic acid, using real-time reverse transcriptase-polymerase chain reaction (RT-PCR) according to the steps described below.

#### RNA Extraction

Isolated bacteria were inoculated in two tubes containing 2 ml LB broth with and without sub-MIC of ascorbic acid. The tubes were incubated at 37°C with shaking at 200 runs per minute (rpm) for 24 h. Bacterial RNA was extracted by the Direct-zol RNA extraction kit (CORP, Australia) according to the kit protocol. Absorbance was measured by a spectrophotometer (Genova, United States), and the ratio of absorbance at 260 nm and 280 nm was used to evaluate the purity of the extracted RNA. A result within the range of 1.8–2 was considered as acceptable purity. The quality of the extracted RNA was assessed *via* gel electrophoresis at 100 V for 60 min.

#### Real-Time Polymerase Chain Reaction

Quantitative RT-PCR was done using one-step SYBR green kits (SensiFAST SYBR Lo-ROX Kit, United Kingdom) in an ABI 7500 instrument (Applied Biosystems, United States). The RT-PCR reaction was prepared with a final volume of 20 μl (master mix: 10 μl, forward primer: 1 μl, reverse primer: 1 μl, reverse transcriptase: 0.2 μl, RNase inhibitor: 0.4 μl, water up to 16 μl, and template: 4 μl). Different genes and primers are listed in Table 1. Negative control samples containing deionized water instead of template, one for each gene, were included in the same PCR run.

**TABLE 1:** Sequence of primers used for RT-PCR.

Gene	Primer	References
*ampC*	F:TCG CCT GAA GGC ACT GGT R: GGT GGC GGT GAA GGT CTT G	Konings et al., 2013
*bla*_*SHV*_	F: CGCCTGTGTATTATCTCCCT R: CGAGTAGTCCACCAGATCCT	Abdelraheem, 2019
*bla*_*TEM*_	F: TTTCGTGTCGCCCTTATTCC R: ATCGTTGTCAGAAGTAAGTTGG	
*gyrA*	F: GTGTGCTTTATGCCATGAG R: GGTTTCCTTTTCCAGGTC	Gorgani et al., 2009
*aacC1*	F: ATGGGCATCATTCGCACATGTAGG R: TTAGGTGGCGGTACTTGGGTC	Aliakbarzade et al., 2014
*lasR*	F: AAGTGGAAAATTGGAGTGGAG R: GTAGTTGCCGACGATGAAG	Abdelraheem and Mohamed, 2021
*lecA*	F: CACCATTGTGTTTCCTGGCGTTCA R: AGAAGGCAACGTCGACTCGTTGAT	
*pelA*	F: AAGAACGGATGGCTGAAGG R: TTCCTCACCTCGGTCTCG	
*16s rRNA*	F:ACG CAA CTG ACGAGT GTG AC R: GAT CGC GAC ACCGAA CTA AT	Abdelraheem et al., 2020

*F, forward; R, reverse.*

*16s rRNA is the reference gene for P. aeruginosa.*

We analyzed the PCR results with relative quantification to *16srRNA* as a reference gene. We calculated the fold changes of mRNA levels using the comparative cycle threshold (ΔΔCt). ΔCt = mean Ct of gene of interest – mean Ct reference gene. ΔΔCt = ΔCT of the test sample –Δ CT of the control sample. The relative quantity (RQ) = 2^–ΔΔ*CT*^ (Livak and Schmittgen, 2001). The relative expression was then analyzed by using free data analysis tools. PCR products were examined by gel electrophoresis to exclude any unspecific products that may be present.

### *In vivo* Assessment of the Antibacterial Effect of Ascorbic Acid

The experiment was conducted according to the standard practices concerning the ethics and animal procedures of the Institutional Research Ethics Committee.

#### Rats

Forty male Wistar rats weighing 250–280 g were obtained from National Research Center (Giza, Egypt). The 40 rats were divided into four groups (10 rats each), group 1 (Pneumonia; PN), group 2 (Vitamin C; VIT C), group 3 (Antibiotic; AB), and group 4 (VIT C+AB). The rats in groups 1, 2, 3, and 4 were intraperitoneally injected with *P. aeruginosa* clinical isolate. Three hours after injection, five rats were culled from group 1 to ensure that pneumonia had been established. Group 1 did not receive any treatment. Treatment options, given by intraperitoneal injection, were started 3 h after inoculation for groups 2, 3, and 4 for 72 h. Group 2 was treated with ascorbic acid (400 mg/kg/day), group 3 was treated with ceftazidime antibiotic (80 mg/kg/day), and group 4 was treated with ceftazidime (20 mg/kg/day) and ascorbic acid (200 mg/kg/day). The dose was calculated according to MIC results and rat pharmacokinetics.

#### Intraperitoneal *P. aeruginosa* Inoculation and Sample Collections

For intraperitoneal (*i.p*) inoculation in rats, the fresh cultured broth was prepared from isolated colonies of *P. aeruginosa*. Rats were injected with *P. aeruginosa* in 1,000 μl saline. The actual inoculum was quantitated by plating 10-fold serial dilutions on agar plates on brain-heart infusion (BHI). The clinical appearance of the rats was followed up. At the end of our experiment, the rats were culled. Initially, the rats were anesthetized, and then the peritoneal cavity and trachea were lavaged with 3 ml of phosphate-buffered saline. Blood samples were collected from the abdominal aorta for bacterial culture or serum separation. The lungs were aseptically removed, divided, and used for bacteriological, biochemical, and histological examination.

#### Bacterial Count

The lungs were aseptically homogenized in sterile saline using tissue homogenizers. A volume of 100 μl of 10-fold serial dilutions of the tissue homogenate, whole blood, bronchoalveolar lavage, and peritoneal lavage fluid were cultured on solid media. The bacterial count was calculated per 1 ml of blood and lavage fluid or per gram of tissue.

#### Quantification of Oxidative Stress Parameters

Blood was centrifuged at 3,000 rpm for 10 min (T30 centrifuge, Germany). Sera were kept frozen at −80°C for further analysis. The lungs were washed with saline and then divided. Specimens were homogenized in ice-cold phosphate buffer, centrifuged for 15 min at 5,000 rpm, and the supernatant was kept frozen at −80°C for further examination. The index of lipid peroxidation is the malondialdehyde (MDA) level, which was determined following the method of Buege and Aust, 1978. Glutathione (GSH) (Moron et al., 1979) and total antioxidant capacity (TAC) were evaluated using colorimetric kits and following the manufacturer’s guidelines.

### Statistical Analyses

Statistical analyses were performed using the Graph Pad Prism, Version 8.0 for Windows (Graph Pad Software, San Diego, CA, United States). Data are presented as means ± standard error of the mean (SEM). The quantitative measurements were assessed by Student’s *t*-test or one-way ANOVA followed by Turkey’s multiple comparison tests, when appropriate. The difference was considered significant when *p*-value < 0.05.

## Results

### *In vitro* Antibacterial and Anti-biofilm Effect of Ascorbic Acid

**FIGURE 1:**
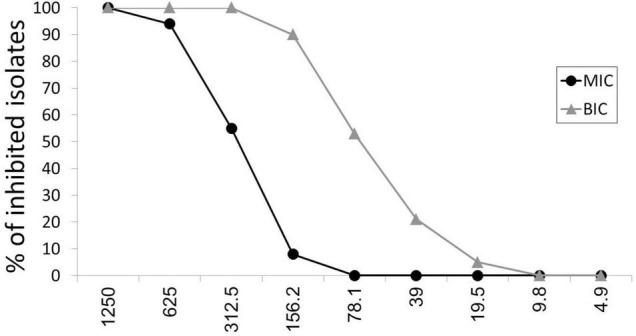
Antibacterial and anti-biofilm effect of ascorbic acid. MIC, minimal inhibitory concentration; BIC, biofilm inhibitory concentration.

Minimal inhibitory concentrations of ascorbic acid among *P. aeruginosa* isolates were 156–1,250 μg/ml, as presented in Figure 1. The MIC50 and MIC90 of ascorbic acid, which inhibit the growth of 50% and 90% of isolates, were 312.5 and 625 μg/ml, respectively.

At sub-inhibitory concentrations (19.5–312.5 μg/ml), ascorbic acid had 100% biofilm inhibitory effect as presented in Figure 1. BIC50 and BIC90, biofilm inhibitory concentrations of ascorbic acid that inhibits the biofilm formation in 50% and 90% of isolates, were 78.1 and 156.2 μg/ml, respectively.

### The Results of Ascorbic Acid/Antimicrobial Combinations

The results of the combination studies are shown in Table 2. Synergy was detected in the five antimicrobial–ascorbic acid combinations tested.

**TABLE 2:** The results of ascorbic acid/antimicrobial combinations.

Antibiotic	MIC^1^	MIC^2^	Number of isolates	FIC
Piperacillin	512	256	14	0.5
	128	64	11	
	32	16	11	
	16	8	7	
	8	4	5	
	4	2	2	
Ceftazidime	128	32	11	0.25
	64	16	9	
	16	4	11	
	4	1	14	
	2	0.5	3	
	1	0.25	2	
Ciprofloxacin	256	32	10	0.125
	32	4	12	
	16	2	10	
	4	0.5	4	
	1	0.125	6	
	0.5	0.06	8	
Gentamicin	128	16	16	0.125
	64	8	4	
	16	2	11	
	8	1	9	
	2	0.25	4	
	1	0.125	6	
Pepracillin/tazobactam	64/4	32/4	5	0.5
	32/4	16/4	16	
	16/4	8/4	8	
	8/4	4/4	12	
	2/4	1/4	6	
	1/4	0.5/4	3	

*^1^MIC of antibiotic alone,^2^MIC of antimicrobial with sub-MIC of ascorbic acid.*

Piperacillin and piperacillin/tazobactam MIC showed onefold decrease when combined with ascorbic acid (FIC = 0.5). Ceftazidime MIC showed twofold reductions when combined with ascorbic acid (FIC = 0.25). Ciprofloxacin and gentamicin MIC showed threefold decreases when combined with ascorbic acid (FIC = 0.125).

### Time-Kill Kinetics Study

**FIGURE 2:**
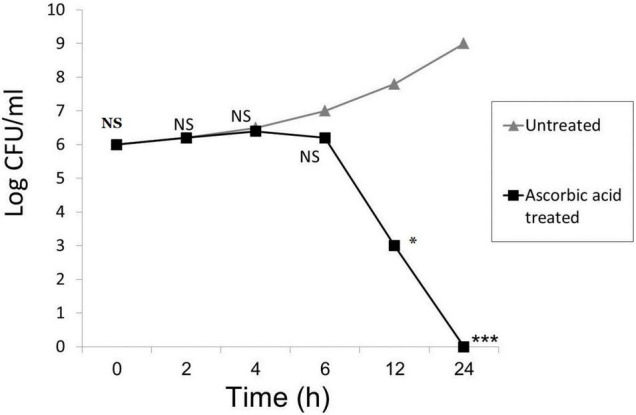
Time-kill kinetics of ascorbic acid. Mean from 3 replicates plotted for all panels; (* means significant, *p*- value* < 0.05, *** < 0.001, and NS means not significant).

The time-kill kinetics study of ascorbic acid against the tested *P. aeruginosa* isolates significantly reduced the number of viable bacterial cells over the first 12, and 24 h, respectively, as shown in Figure 2.

### Effect of Ascorbic Acid on Gene Expression

**FIGURE 3:**
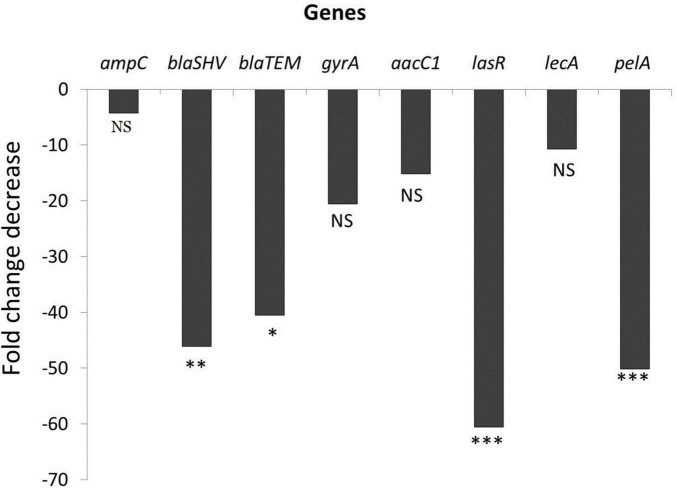
Effect of ascorbic acid on gene expression (* means significant, *p*- value* < 0.05, value** < 0.01, *** < 0.001).

The results showed that the expression levels of all tested antimicrobial resistance genes (*ampC, bla_*SHV*_, bla_*TEM*_, gyrA*, and *aacC1*) and biofilm-associated gnes (*lasR, lecA*, and *pelA*) were down-regulated as shown in Figure 3. We observed a 46.1- and 40.5-fold decrease in the expression of the *bla*_*SHV*_ and *bla*_*TEM*_ genes, respectively, under ascorbic acid-treated condition (*p* value < 0.05). Results also confirmed that there was a significant fold change decrease (60.6 and 50.1) in transcription of biofilm-associated genes, *lasR* and *pelA*, respectively, (*p* value < 0.05) compared to untreated culture, thus confirming the anti-biofilm effect of ascorbic acid.

### *In vivo* Assessment of the Antibacterial Effect of Ascorbic Acid

All remaining rats in group 1 died after 24 h, and then all the rats were culled after 72 h (6 h after the last dose of treatment).

#### Bacterial Count

**FIGURE 4:**
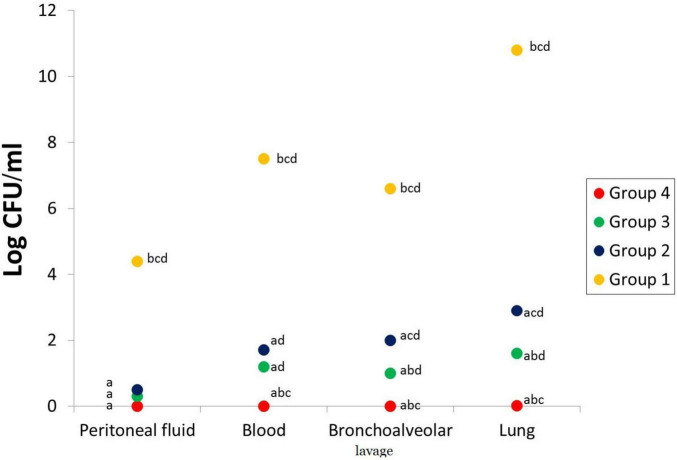
Bacterial count in the peritoneal lavage, blood, bronchoalveolar lavage, and lungs of different rat groups. ^a^Significant difference compared to PN group. ^b^Significant difference compared to VIT C group. ^c^Significant difference compared to AB group. ^d^Significant difference compared to VIT C+AB group. PN, pneumonia; VIT C, vitamin C; AB, antibiotic.

The five rats culled at the start of the experiment (3 h after inoculation of *P. aeruginosa*) presented bilateral pneumonia with a mean *P. aeruginosa* count of log_10_; 4.4, 7.5, 6.6, and 10.8 for peritoneal fluid, blood, bronchoalveolar lavage, and lung, respectively (Figure 4). The viable count after treatment was calculated in peritoneal fluid, blood, bronchoalveolar lavage, and lung, and the mean is presented in Figure 4. VIT C+AB group shows complete eradication of *P. aeruginosa* infection. A significant reduction of the bacterial count was seen in the AB group and VIT C group, respectively, in comparison with the untreated PN group (*p* value < 0.05).

#### Oxidative Stress Parameters

**FIGURE 5:**
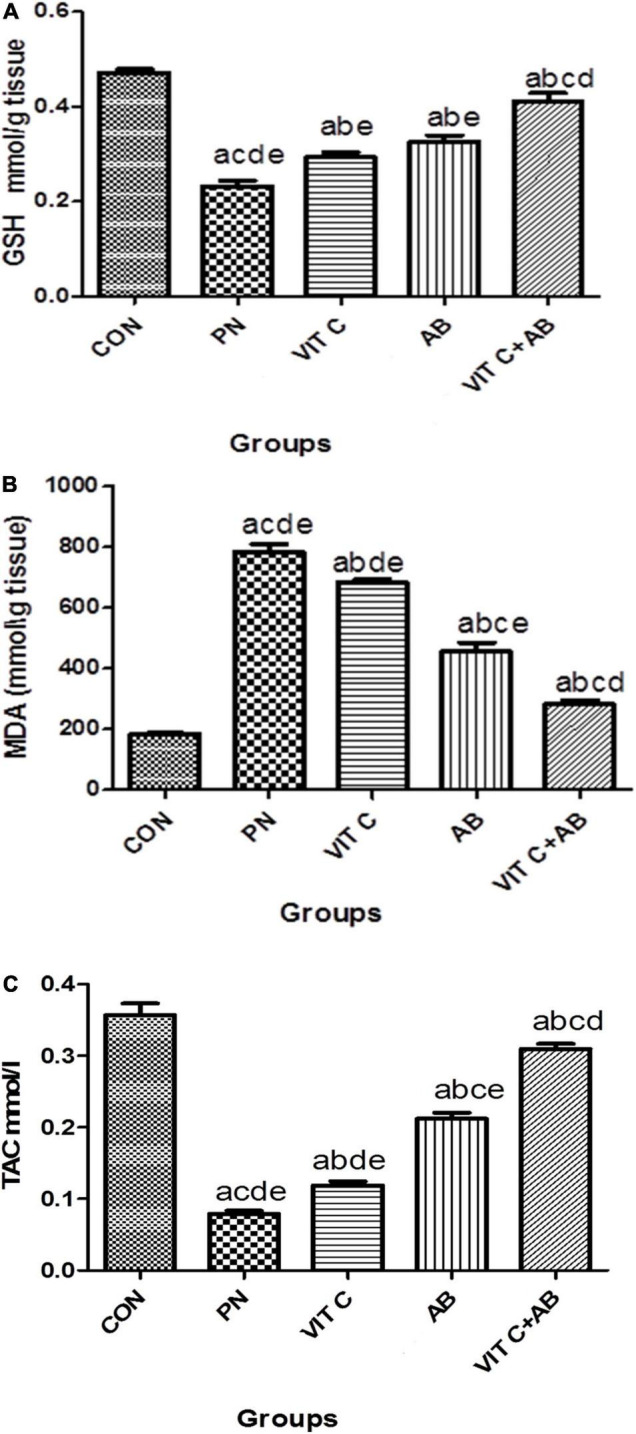
Oxidative stress parameters of different rat groups. **(A)** Glutathione (GSH) in lung tissue, **(B)** malondialdehyde (MDA) in lung tissue, **(C)** total antioxidant capacity (TAC) in serum. ^a^Significant difference compared to PN group. ^b^Significant difference compared to VIT C group. ^c^Significant difference compared to AB group. ^d^Significant difference compared to VIT C+AB group. PN, pneumonia group; VIT C, vitamin C; AB, antibiotic.

VIT C alone, AB alone, and VIT C+AB groups showed a significant decrease in MDA with a substantial increase in GSH and TAC compared to the control group. Co-administration of VIT C + AB showed more improvement and normalization of oxidative stress parameters compared to VIT C or AB groups, as shown in Figures 5A–C.

#### Histological Examination

**FIGURE 6:**
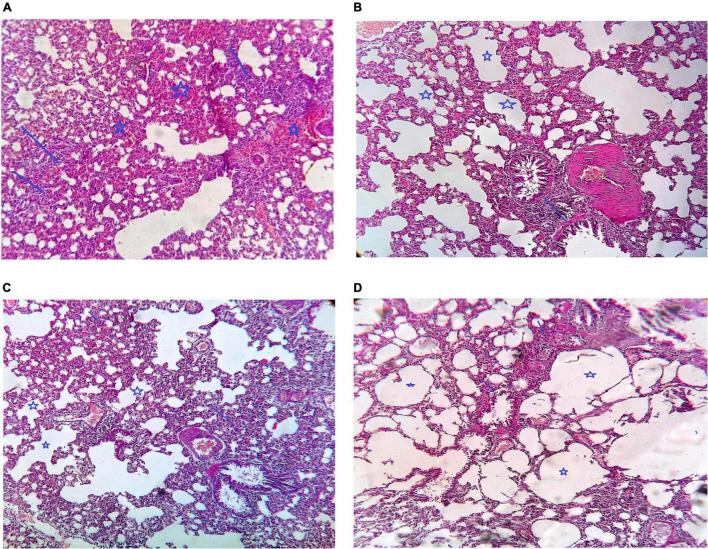
Histological examination of different groups of rat lungs. **(A)** PN group, Arrow show multiple foci of moderate inflammatory infiltrate. Star showed marked intraalveolar congestion, **(B)** VIT C group: Arrow showed that there is alveoli are still congested and contain inflammatory infiltrate but less than that in case of pneumonia only. Star showed that there is some alveoli are cleared from congestion and inflammatory infiltrate, **(C)** AB group: Arrow show moderate peribroncheal inflammatory infiltrate. Star show alveoli cleared from congestion and inflammatory infiltrate, **(D)** VIT C+ AB group: Arrow showed there is no peribronchial inflammatory infiltrate. Star showed cleared alveoli with no congestion or inflammation.

Comparison of the histological picture of rat lung tissues of different groups is presented in Figures 6A–D. The five rats killed at the start of the experiment (PN group) showed a histological picture of bilateral pneumonia. Improvement of the histological picture in all treatment groups was detected but with different degrees. The VIT C+AB group showed nearly complete improvement of the signs of pneumonia. Signs of pneumonia were still present in VIT C and AB groups, respectively, but less than PN group.

## Discussion

This study is the first insight wherein we evaluated antimicrobial and anti-biofilm effect of vitamin C against *P. aeruginosa* clinical isolates *in vitro* and *in vivo*.

The antimicrobial effect of vitamin C exists against the all the *P. aeruginosa* clinical isolates tested. To our surprise, vitamin C inhibited the growth of *P. aeruginosa* clinical isolates at lower concentration ranges of 156.2–1,250 μg/ml, while MIC50 and MIC90 were 312.5 and 625 μg/ml, respectively.

The antimicrobial effect of vitamin C corroborates with the previous results by Golonka et al. (2017), which depict that the MIC 310 μg/mL of vitamin C could inhibit *P. aeruginosa* growth *in vitro*. In addition, vitamin C at low concentration (150 μg/ml) was shown to inhibit the growth of *S. aureus* and *E. faecalis* (Verghese et al., 2017). Further studies reported the antibacterial effect of vitamin C against *E. coli* and Klebsiella (Abd El-Baky and El-Gebaly, 2012).

This is in contrast to the findings reported by Pandit et al., 2017. They reported that vitamin C at low concentrations did not exhibit any inhibitory effect against planktonic bacteria. They suggested that vitamin C enhances the antibiotic bacterial susceptibility without demonstrating direct bactericidal activity.

With regard to the reports mentioned above, the differences of low or no antibacterial effect of vitamin C are probably attributed to the variations in the methodologies attributed during the study, i.e., differences in the bacterial strains, bacterial cell density, culture media composition, and concentration of vitamin C.

Moving on to the biofilm production, we found an anti-biofilm effect of vitamin C on the same isolates that were examined, based on the absorbance results that we obtained from the ELISA reader. Biofilm inhibitory concentrations of ascorbic acid among *P. aeruginosa* isolates were 19.5–312.5 μg/ml, with BIC50 and BIC90 being 78.1 and 156.2 μg/ml, respectively. In this aspect, Abd El-Baky and El-Gebaly (2012) reported that vitamin C at a concentration of 100 mg/ml could inhibit biofilm production on the catheter surface. There was up to a 92% reduction in biofilm production. Likewise, Sendamangalam et al. (2011) and Eydou et al. (2020) reported 2 mg/ml and 5.61 mg/ml, respectively, as the biofilm inhibitory concentration of vitamin C against *S. mutans.*

Furthermore, vitamin C could effectively counteract biofilm production by methicillin-resistant *S. aureus* (MRSA) at a lower concentration range of 8–16 μg/ml (Ali Mirani et al., 2018).

A synergistic effect was detected in the five antimicrobial–ascorbic acid combinations tested; piperacillin and piperacillin/tazobactam (FIC = 0.5), ceftazidime (FIC = 0.25), ciprofloxacin and gentamicin (FIC = 0.125). The enhancement of antibiotic activity or the reversal of antibiotic resistance by non-traditional antibiotics support the classification of vitamin C as a modifier of antibiotic activity. Similar synergistic effects between antimicrobials and ascorbic acid were previously reported by Cursino et al. (2005) and Abd El-Baky and El-Gebaly (2012).

The synergistic effect of ascorbic acid with antibiotics may be due to its effect on certain metabolic activities associated with protein synthesis inside bacterial cells, making the bacterial cells more permeable to antibiotics through its effect on the cytoplasmic membrane, or it could be due to the effect of hydrogen peroxide produced by the oxidation of ascorbic acid, which causes antibiotics to have a higher potency (Kramarenko et al., 2007). Certain antibiotics increase the production of reactive oxygen species (free radicals) in various bacterial species, suggesting that ascorbic acid may serve as a free radical scavenger (Albesa et al., 2004). Also, the synergistic effect of ascorbic acid with antibiotics may be due to down regulation of antibiotic-resistant genes.

Biofilm-forming *P. aeruginosa* strains demonstrated higher expression levels of *lasR, lecA*, and *pelA* genes (Abdelraheem et al., 2020).

Further, to confirm the anti-biofilm effect of vitamin C and the corresponding synergistic effect with antibiotics against *P. aeruginosa* tested isolates, a transcriptional response was analyzed in the presence of Vitamin C. The results showed that all tested biofilm-associated gene expression levels vital to *P. aeruginosa* biofilm formation (*lasR, lecA*, and *pelA*) were down-regulated after being treated with ascorbic acid, with significant decrease in *lasR and pelA* (*p* value < 0.05). Therefore, it was suggested that the anti-biofilm activity of vitamin C was due to the inhibition of biofilm-forming genes expression.

Also, the expression levels of all tested genes (*ampC, bla_*SHV*_, bla_*TEM*_, aacC1, and gyrA*) are essential for resistance to piperacillin, piperacillin/tazobactam, ceftazidime, ciprofloxacin and gentamicin were down-regulated after treating *P. aeruginosa* isolates with ascorbic acid, the expression levels of all tested genes (ampC, bla_*SHV*_, bla_*TEM*_, aacC1, and gyrA), which are essential for resistance to piperacillin, piperacillin/tazobactam, ceftazidime, ciprofloxacin and gentamicin, were down-regulated, with a significant decrease in bla_*SHV*_ and bla_*TEM*_ (*P* value < 0.05).

In our *in vivo* investigation, the efficacy of vitamin C treatment in rats after infection with *P. aeruginosa* showed that the administration of vitamin C in the infected animal partially resolved the disease and lowered the bacterial count. *In vivo*, the rats’ vitamin C and antibiotic assessment showed that antibiotic alone has a better antibacterial effect than vitamin C alone. Still, the administration of both at a lower dose for treating pseudomonas infection in rats has a synergistic and more powerful impact. Vitamin C shows excellent *in vitro* results as an antibacterial and anti-biofilm agent. Still, a combination of vitamin C and antibiotics is necessary to eradicate the bacterial infection *in vivo.* Vitamin C has a synergistic effect with antibiotics *in vitro* and *in vivo*. Vitamin C should be routinely prescribed with antibiotics to treat pseudomonas infections in clinical settings as this combination will shorten the antibiotic course, decrease the dose of the antibiotic, and reduce the development of bacterial resistance. However, further *in vivo* studies are needed before generalizing the concept about the effectiveness of ascorbic acid–antibiotics combined therapy. Future research on ascorbic acid–antimicrobial interactions is required to control MDR bacteria.

## Data Availability Statement

The original contributions presented in the study are included in the article/supplementary material, further inquiries can be directed to the corresponding author/s.

## Ethics Statement

The studies involving human participants were reviewed and approved by Ethical Committee of Minia University. The patients/participants provided their written informed consent to participate in this study. The animal study was reviewed and approved by Institutional Research Ethics Committee.

## Author Contributions

WA, RR, AA, and YM: concept and study design. WA, MR, and RY: experiments and results analysis. All authors drafted and revised the manuscript and read and approved the final manuscript.

## Conflict of Interest

The authors declare that the research was conducted in the absence of any commercial or financial relationships that could be construed as a potential conflict of interest.

## Publisher’s Note

All claims expressed in this article are solely those of the authors and do not necessarily represent those of their affiliated organizations, or those of the publisher, the editors and the reviewers. Any product that may be evaluated in this article, or claim that may be made by its manufacturer, is not guaranteed or endorsed by the publisher.
